# Effect of interferon beta-1a subcutaneously three times weekly on clinical and radiological measures and no evidence of disease activity status in patients with relapsing–remitting multiple sclerosis at year 1

**DOI:** 10.1186/s12883-018-1145-x

**Published:** 2018-09-14

**Authors:** Anthony Traboulsee, David K. B. Li, Mark Cascione, Juanzhi Fang, Fernando Dangond, Aaron Miller

**Affiliations:** 1 University of British Columbia, S113-2211 Wesbrook Mall, Vancouver, BC V6T 1Z7 Canada; 2 Tampa Neurology Associates, South Tampa Multiple Sclerosis Center, 2919 W. Swann Avenue, Suite 401, South Tampa, FL 33609 USA; 3 EMD Serono, Inc., One Technology Place, Rockland, MA 02370 USA; 4 EMD Serono, Inc, 45A Middlesex Tpke, Billerica, MA 01821 USA; 5 Mount Sinai Hospital, 5 East 98th Street, 1st Floor, New York, NY 10029 USA

**Keywords:** Relapsing–remitting multiple sclerosis, Clinical trials, Interferon-beta subcutaneously, Disability progression, MRI, No evidence of disease activity

## Abstract

**Background:**

In the PRISMS study, interferon beta-1a subcutaneously (IFN β-1a SC) reduced clinical and radiological disease burden at 2 years in patients with relapsing–remitting multiple sclerosis. The study aimed to characterize efficacy of IFN β-1a SC 44 μg and 22 μg three times weekly (tiw) at Year 1.

**Methods:**

Exploratory endpoints included annualized relapse rate (ARR), 3-month confirmed disability progression (1-point Expanded Disability Status Scale increase if baseline was < 6.0 [0.5-point if baseline was ≥6.0]), active T2 lesions, and no evidence of disease activity (NEDA; defined as no relapses [subanalyzed by relapse severity], 3-month confirmed progression, or active T2 lesions). Effect of IFN β-1a SC in prespecified patient subgroups was also assessed.

**Results:**

Patients were randomized to IFN β-1a 22 μg (*n* = 189), 44 μg (*n* = 184), or placebo (*n* = 187). At 1 year, IFN β-1a SC tiw reduced ARR (*p* < 0.001), risk of disability progression (*p* ≤ 0.029), and mean number of active T2 lesions per patients per scan (*p* < 0.001) versus placebo. Clinical and radiological benefits were seen as early as Month 2 and 3. Outcomes in subgroups were consistent with those in the overall population. More patients treated with IFN β-1a SC tiw achieved NEDA status, versus placebo, regardless of relapse severity (*p* ≤ 0.006).

**Conclusion:**

Clinical, radiological, and NEDA outcomes at Year 1 were consistent with Year 2 results. Treatment efficacy was consistent in pre-specified patient subgroups.

**Electronic supplementary material:**

The online version of this article (10.1186/s12883-018-1145-x) contains supplementary material, which is available to authorized users.

## Background

PRISMS (Prevention of Relapses and disability by Interferon beta-1a Subcutaneously in Multiple Sclerosis) was a 2-year, double-blind, placebo-controlled study in patients with relapsing–remitting multiple sclerosis (RRMS), which demonstrated that interferon beta-1a (IFN β-1a) subcutaneously (SC) three times weekly (tiw) significantly reduced the number of relapses, risk of 3-month confirmed disability progression, and number of active T2 lesions, compared with placebo [1]. In the 2-year extension phase of PRISMS, these clinical and radiological benefits were sustained following continuous IFN β-1a SC tiw therapy [2].

While clinical trials evaluating disease-modifying drugs for the treatment of RRMS typically last 2 years or more, some recent trials have been designed to evaluate outcomes over 1 year; additionally, a recent cohort study has found that no evidence of disease activity (NEDA) status at 1 year predicts a lack of disability progression at 7 years [3–5]. The current post hoc analyses were conducted to characterize the efficacy of IFN β-1a SC tiw compared with placebo on clinical and radiological endpoints, and NEDA, during the first year of the PRISMS study. Additional subgroup analyses were conducted to assess the relationship between baseline and clinical characteristics and the treatment effect of IFN β-1a SC tiw on NEDA endpoints, and the impact of relapse severity on NEDA.

## Methods

### Study design and treatment

The full details of the PRISMS-2 study have been published previously [1]. Eligible patients (18–50 years of age) had clinically definite or laboratory-supported definite RRMS based on the Poser criteria [6], a history of two or more relapses in the previous 2 years, and an Expanded Disability Status Scale (EDSS) score of 0–5.0. Patients were assigned randomly (1:1:1) to IFN β-1a 44 or 22 μg SC tiw or placebo for 2 years. The amount of study drug administered was gradually increased (titrated) to the full dose at the beginning of treatment: patients received 20% of their assigned dose for 2–4 weeks, followed by 50% of this dose for another 2–4 weeks, before finally receiving the full dose.

Patients underwent neurological assessments every 3 months, and as needed for relapse assessment. All patients had magnetic resonance imaging (MRI) scans biannually (cohort 1), and a subset of patients had monthly proton density (PD)/T2 and T1 gadolinium- enhancing (Gd+) scans prior to treatment initiation and during the first 9 months of treatment (cohort 2).

Relapses were defined as a new or worsening symptom attributable to MS, accompanied by an appropriate new neurological abnormality or focal neurological dysfunction lasting at least 24 h in the absence of fever, and preceded by stability or improvement for at least 30 days [1]. A visit to the study center within 7 days of relapse for confirmation and assessment of severity by the assessing neurologist was requested. Relapse severity was categorized according to quantitative changes in the Scripps Neurological Rating Scale (NRS) score, whereby the worst score during the relapse was compared with the patient’s score prior to the start of the relapse: a decrease of 0–7 points was defined as mild, 8–14 as moderate, and ≥ 15 as severe. If it was not possible to evaluate a relapse using the Scripps NRS at the time of worst severity, the relapse was scored according to its effect on activities of daily living. All relapses, as defined by the study protocol, were reported.

Post hoc analyses examined between-treatment differences (IFN β-1a SC tiw compared with placebo) in the following clinical endpoints up to Year 1: annualized relapse rate (ARR); risk of 3-month confirmed disability progression (1-point increase in EDSS score if the baseline EDSS score was < 6.0, or 0.5-point increase if the baseline EDSS score was ≥6.0, with the increase being confirmed at a visit 3 months later); time to first relapse; and proportion of patients relapse-free over 3, 6, 9, and 12 months. The radiological endpoints assessed up to Year 1 included: mean number of active T2 lesions (defined as a new or newly enlarging lesion, or a recurrent lesion [‘recurrent’ lesions were those that appeared on one scan, were not present on the next scan, then appeared again on a third scan]) per patient per scan (total study cohort) [7]; monthly percentages of patients free of Gd + lesions; and cumulative mean numbers of active T2, Gd+, and combined unique active lesions (defined as an active lesion on T1 post-Gd, T2 sequences, or both, avoiding double counting) per patient per scan in the frequent-MRI cohort.

Further analyses assessed clinical and radiological efficacy results at Year 1 in patient subgroups stratified by prespecified baseline characteristics, including age (< 40 vs. ≥40 years), sex (male vs. female), baseline EDSS score (≤median vs. >median; median baseline EDSS score: 2.5), baseline number of relapses (< 3 vs. ≥3), baseline burden of disease (BOD; ≤median vs. >median; median baseline burden of disease: 1992.5 mm^2^), and time since MS onset (< 4 vs. ≥4 years).

Treatment differences at Year 1 were examined across a range of composite endpoints. These endpoints included the proportion of patients who had no evidence of clinical disease activity (defined as no protocol-defined relapses and no 3-month confirmed disability progression); were free of active T2 lesions; and achieved NEDA, defined as clinical activity free and no active T2 lesions. NEDA results were also analyzed using Scripps NRS score–assessed relapse definitions based on moderate and/or severe relapse and severe relapse criteria.

### Statistical analyses

Comparison of ARR between treatment groups was based on a negative binomial model adjusting for baseline EDSS score (≤3.5 vs. > 3.5), age (< 40 vs. ≥40 years), number of relapses in 2 years prior to screening, and baseline T2 BOD (total area [mm^2^] of all T2 lesions, outlined on the PD/T2 scan) with log time on study up to 1 year as an offset variable.

Treatment differences in the proportions of patients free from relapse (cumulative assessment up to 3, 6, 9, and 12 months) were examined using a logistic model adjusting for treatment center.

Comparison of mean number of active T2 lesions per patient per scan (Month 6 and Year 1) was based on a negative binomial model adjusting for baseline BOD, and treatment center with log number of MRI scans up to Year 1 as an offset variable.

Between-treatment comparisons of no evidence of clinical disease activity and NEDA endpoints were based on adjusted logistic models.

## Results

### Patients

The intent-to-treat population in PRISMS comprised 560 patients who had been randomly assigned to receive IFN β-1a 22 μg (*n* = 189) or 44 μg (*n* = 184) SC tiw, or placebo (*n* = 187). The monthly MRI cohort (cohort 2) comprised 205 patients who had been treated with IFN β-1a 22 μg or 44 μg SC tiw; or placebo (67, 68, and 70 randomized patients, respectively). Baseline demographic and clinical characteristics were similar across treatment groups (see Additional file 1: Table S1) [1].

### Efficacy up to year 1

#### Relapses

**Fig. 1:**
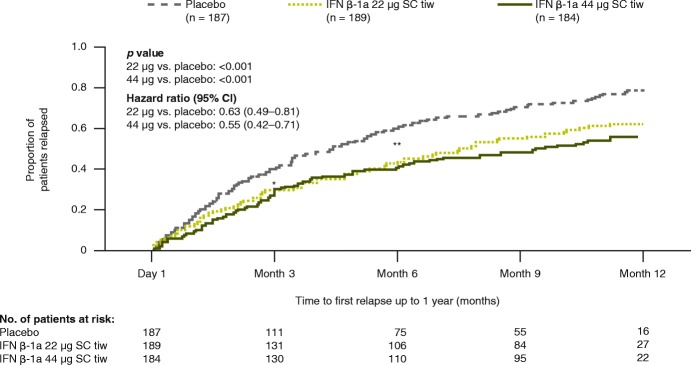
Time to first relapse over 1 year. Assessed using Cox’s proportional hazards model adjusting for baseline EDSS score (≤3.5 vs. > 3.5), age (< 40 vs. ≥40 years), baseline number of relapses, and baseline burden of disease (total area [mm^2^] of all MS lesions, outlined on the PD/T2 scan). A significant difference compared with placebo was seen from Month 3 (*p < 0.05) onward with IFN β-1a 44 μg SC tiw, and from Month 6 (**p < 0.01) onward with IFN β-1a 22 μg SC tiw. CI: confidence interval; EDSS: Expanded Disability Status Scale; IFN β-1a: interferon beta-1a; MS: multiple sclerosis; PD: proton density; SC: subcutaneously; tiw: three times weekly

Treatment with both IFN β-1a 44 and 22 μg SC tiw reduced clinical and MRI disease activity compared with placebo at Year 1. At Year 1, adjusted mean (95% confidence interval [CI]) ARRs were lower in patients treated with IFN β-1a 44 μg (0.92 [0.78–1.09]) and 22 μg (1.01 [0.86–1.19]) SC tiw compared with placebo (1.49 [1.29–1.72]), representing reductions of 38% and 32%, respectively (both *p* < 0.001). Compared with placebo, time to first relapse over 1 year was significantly delayed by IFN β-1a 44 and 22 μg SC tiw treatment (*p* < 0.001, Fig. 1). Increases in the proportion of IFN β-1a SC patients relapse free (compared with placebo) were significant beginning at Month 3 (71.7% vs. 60.8%; *p* = 0.0230) through Month 12 with IFN β-1a 44 μg SC tiw, and from Month 6 (56.5% vs. 40.3%; *p* = 0.0019) through Month 12 with IFN β-1a 22 μg SC tiw (Fig. 1). IFN β-1a 44 μg SC tiw also significantly reduced the mean cumulative number of relapses compared with placebo during each incremental 3-month period in Year 1, including at 0–3 months (0.30 vs. 0.43; *p* = 0.0421) and at > 3–6, > 6–9, and > 9–12 months (*p* < 0.001), while IFN β-1a 22 μg SC tiw significantly reduced mean cumulative relapse number compared with placebo over 6, 9, and 12 months (*p* < 0.01). Incremental relapse counts over each 3-month period up to Year 1 were lower in both IFN β-1a SC tiw groups compared with placebo (Additional file 2: Figure S1).

#### Disability progression

**Fig. 2:**
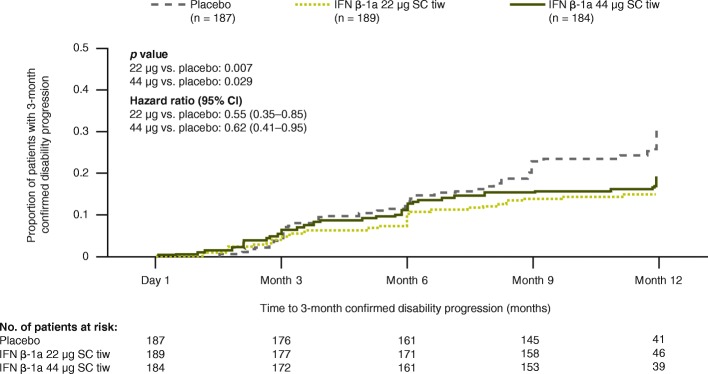
Time to 3-month confirmed disability progression^a^ over 1 year. Assessed using Cox’s proportional hazards model adjusting for baseline EDSS score (≤3.5 vs. > 3.5) and age (< 40 vs. ≥40 years). ^a^EDSS progression was defined as 1-point increase in EDSS score if the baseline EDSS score was < 6.0 or a 0.5-point increase if the baseline EDSS score was ≥6.0. (EDSS scores could be > 5 if scores increased between screening and baseline). CI: confidence interval; EDSS: Expanded Disability Status Scale; IFN β-1a: interferon beta-1a; SC: subcutaneously; tiw: three times weekly

The risk of 3-month confirmed disability progression was significantly reduced with IFN β-1a 44 μg and 22 μg SC tiw over 1 year compared with placebo, showing reductions of 38% and 45%, respectively (Fig. 2).

#### MRI activity

**Fig. 3:**
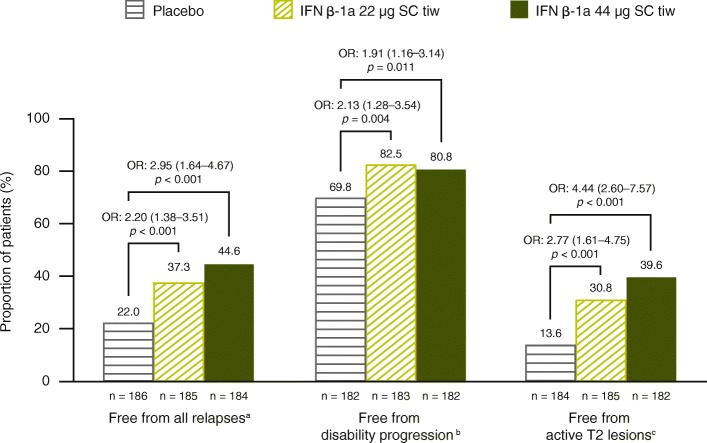
Proportions free from relapses, disability progression, and active T2 lesions up to 1 year. Based on a logistic regression adjusting for age (< 40 vs. ≥40 years), sex, baseline EDSS score (≤3.5 vs. > 3.5), number of relapses prior to the study within 24 months, and time since MS onset; *p* values indicate effect of IFN β-1a SC tiw compared with placebo. Values within parentheses are 95% CI values. Active T2 lesions obtained at 6-month and 1-year scans were included. ^a^Endpoint is missing if the patient withdrew before the first year and did not have any relapses. ^b^Endpoint is missing if the patient withdrew before the first year and did not have 3-month confirmed EDSS progression before withdrawal; EDSS progression was defined as 1-point increase in EDSS score if the baseline EDSS score was < 6.0 or 0.5-point increase if the baseline EDSS score was ≥6.0 (EDSS scores could be > 5 if scores increased between screening and baseline). ^c^Endpoint is missing if the patient withdrew before the first year and did not have any active T2 lesions. CI: confidence interval; EDSS: Expanded Disability Status Scale; IFN β-1a: interferon beta-1a; OR: odds ratio; SC: subcutaneously; tiw: three times weekly

For the entire cohort, IFN β-1a 44 μg and 22 μg SC tiw significantly reduced the mean (standard deviation) number of active T2 lesions per patient per scan compared with placebo over 1 year (1.16 [1.94] and 1.95 [3.41] vs. 3.83 [4.57], respectively; *p* < 0.001 for both comparisons). The proportion of patients free of T2 lesions over 1 year was significantly higher with IFN β-1a 22 μg and 44 μg SC tiw treatment, compared with placebo (*p* < 0.001; Fig. 3). Overall, 26.3%, 49.4%, and 63.9% of patients in the placebo, IFN β-1a 22 μg SC tiw, and IFN β-1a 44 μg SC tiw groups, respectively, were free of active T2 lesions on the scan at 1 year (that is, not considering the scan at 6 months; *p* < 0.001 for both IFN groups compared with placebo). Analyses of data from the frequent, monthly MRI cohort (cohort 2) showed that IFN β-1a SC tiw treatment significantly reduced MRI disease activity compared with placebo from as early as Month 2, as evidenced by a decrease in the mean number of Gd + lesions per patient per scan and an increase in the proportion of patients who were free of Gd + lesions (Additional file 3: Figure S2).

#### Subgroup analysis

**Fig. 4:**
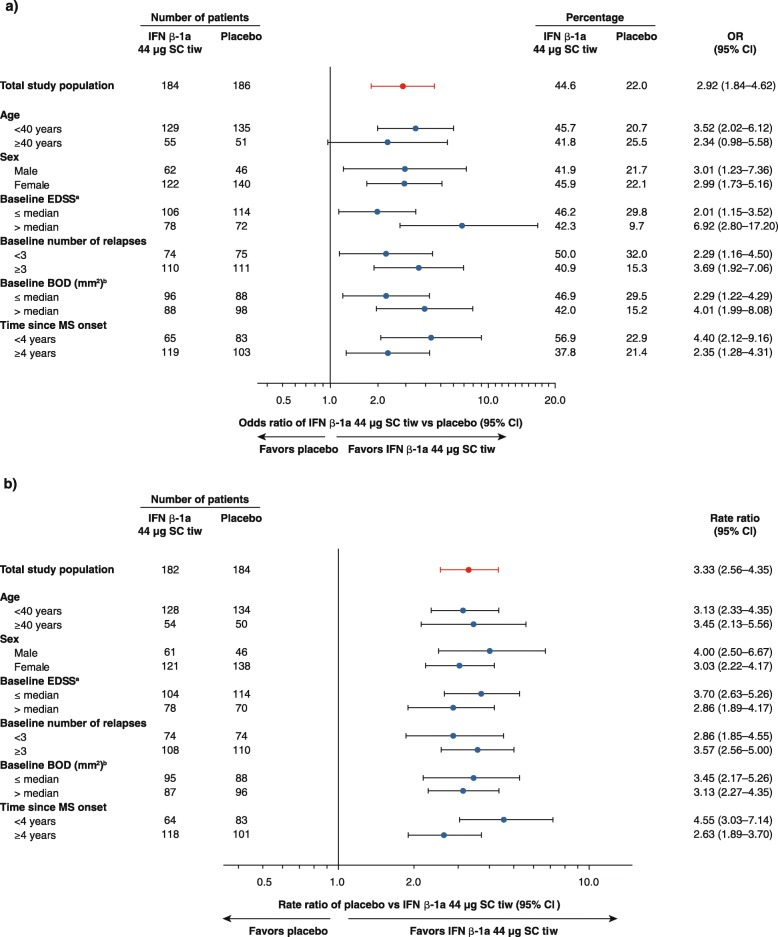
Year 1 effect of IFN β-1a 44 μg SC tiw in prespecified subgroups of patients. **a** ORs of IFN β-1a 44 μg SC tiw compared with placebo for the proportion of patients free from relapse at Year 1. Based on logistic model adjusting for age, number of pre-study relapses, baseline EDSS score, and baseline BOD as covariates. (Age, number of pre-study relapses, baseline EDSS score, and baseline BOD were not covariates used in analysis of the subgroups determined by each of these respective characteristics.) **b** Rate ratios for placebo compared with IFN β-1a 44 μg SC tiw for the number of active T2 lesions up to Year 1. Based on a negative binomial model adjusting for baseline BOD as covariate, and log number of scans up to Year 1 as an offset variable. Baseline BOD was not a covariate used in the analysis of the baseline BOD subgroups. Active T2 lesions up to 1 year included those lesions detected at the 6-month or 1-year MRI assessments. ^a^Median baseline EDSS score: 2.5. ^b^Median baseline BOD: 1992.5 mm^2^. BOD: burden of disease; CI: confidence interval; EDSS: Expanded Disability Status Scale; IFN β-1a: interferon beta-1a; MRI: magnetic resonance imaging; MS: multiple sclerosis; OR: odds ratio; SC: subcutaneously; tiw: three times weekly

Treatment effects on clinical and MRI outcomes in the prespecified patient subgroups were consistent with the overall population at Year 1. In all subgroups, point estimates and 95% CIs indicated treatment benefits in favor of IFN β-1a 44 μg SC tiw compared with placebo on the proportion of patients free from relapses (with the exception of patients aged ≥40 years; Fig. 4a) and number of active T2 lesions (Fig. 4b). A significant benefit of IFN β-1a 44 μg SC tiw treatment compared with placebo with regard to the proportion of patients free from 3-month confirmed disability progression was observed in females (*p* = 0.013) and in patients with BOD above the median level (1992.5 mm^2^; *p* = 0.007). Although there was a consistent trend for estimates of relative treatment effects in favor of IFN β-1a 44 μg SC tiw compared with placebo across all other patient subgroups, it did not reach statistical significance. Significant treatment benefits on the proportion of patients free from relapses over 1 year were also seen in favor of IFN β-1a 22 μg SC tiw compared with placebo in all age and sex subgroups, and in the subgroups of patients with greater disease activity or duration at baseline; significant treatment benefits on the number of active T2 lesions up to 1 year were seen in favor of IFN β-1a 22 μg SC tiw compared with placebo in all prespecified patient subgroups (data not shown).

#### No evidence of clinical disease activity and NEDA outcomes

**Fig. 5:**
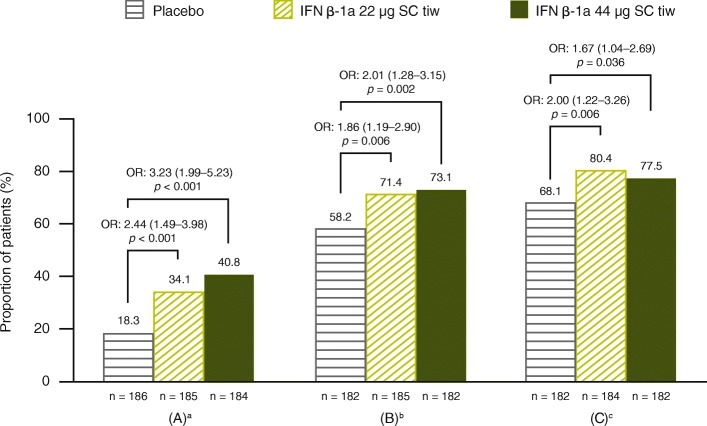
Proportion of patients with no evidence of clinical disease activity. Based on (**a**) absence of all protocol-defined relapses, (**b**) absence of Scripps-assessed moderate and/or severe relapses, or (**c**) absence of severe relapses, at 1 year. Based on a logistic model adjusting for age (< 40 vs. ≥40 years), sex, baseline EDSS (≤3.5 vs. > 3.5), number of relapses in 2 years prior to screening, and time since MS onset. Values within parentheses are 95% CI values. ^a^Defined as no relapses and no 3-month confirmed disability progression (1-point increase in EDSS score if the baseline EDSS score was < 6.0 or 0.5-point increase if the baseline EDSS score was ≥6.0). (EDSS score could be > 5 if scores increase between screening and baseline.) ^b^Defined as no relapses and no 3-month confirmed disability progression (1-point increase in EDSS score if the baseline EDSS score was < 6.0 or 0.5-point increase if the baseline EDSS score was ≥6.0). (EDSS score could be > 5 if scores increase between screening and baseline.) Relapses are defined as Scripps-assessed moderate and/or severe. ^c^Defined as no relapses and no 3-month confirmed disability progression (1-point increase in EDSS score if the baseline EDSS score was < 6.0 or 0.5-point increase if the baseline EDSS score was ≥6.0). (EDSS score could be > 5 if scores increase between screening and baseline.) Relapses are defined as Scripps-assessed severe. EDSS: Expanded Disability Status Scale; IFN β-1a: interferon beta-1a; OR: odds ratio; SC: subcutaneously; tiw: three times weekly

**Fig. 6:**
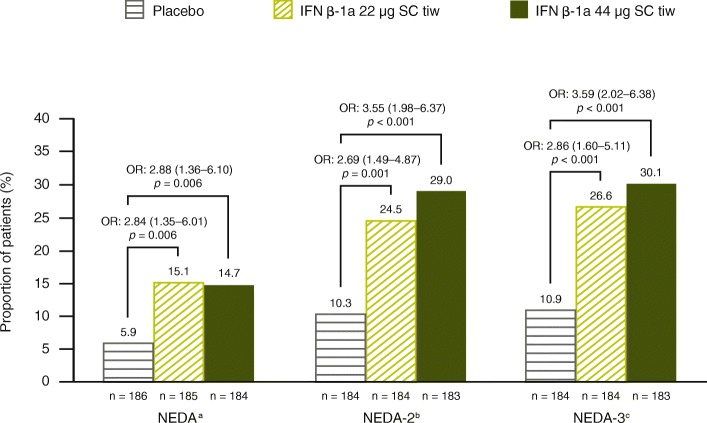
Proportion of patients with NEDA status up to 1 year. Based on a logistic model adjusting for age (< 40 vs. ≥40 years), sex, baseline EDSS (≤3.5 vs. > 3.5), number of relapses in 2 years prior to screening, and time since MS onset. Active T2 lesions obtained at 6-month and 1-year scans were used. ^a^NEDA defined as no relapses, no 3-month confirmed disability progression, and no active T2 lesions over 1 year; disability progression was defined as a 1-point increase in EDSS score if the baseline EDSS score was < 6.0 or 0.5-point increase if the baseline EDSS score was ≥6.0. (EDSS score could be > 5 if scores increase between screening and baseline.). ^b^NEDA-2, defined as NEDA with relapses defined as Scripps-assessed moderate and/or severe. ^c^NEDA-3, defined as NEDA with relapses defined as Scripps-assessed severe. EDSS: Expanded Disability Status Scale; IFN β-1a: interferon beta-1a; NEDA: no evidence of disease activity; OR: odds ratio; SC: subcutaneously; tiw: three times weekly

Compared with placebo, significantly more patients treated with IFN β-1a SC tiw were free from relapses, 3-month confirmed disability progression, and active T2 lesions (free at both 6-month and 1-year MRI scans) over Year 1 (Fig. 3). These treatment effects were reflected in composite outcomes, with significantly more patients treated with IFN β-1a 22 and 44 μg SC tiw achieving no evidence of clinical disease activity (Fig. 5) and NEDA status (Fig. 6) over Year 1, compared with patients receiving placebo.

Across treatment groups, greater proportions of patients achieved NEDA as defined by no Scripps-assessed moderate and/or severe relapse (NEDA-2) and NEDA as defined by no Scripps-assessed severe relapse (NEDA-3) than achieved NEDA as defined by absence of any protocol-defined relapses (Fig. 6). Significant treatment benefits were seen in favor of IFN β-1a 22 and 44 μg SC tiw, regardless of the relapse criteria used. Odds ratios (ORs) for IFN β-1a 44 μg SC tiw versus placebo were larger when the definition of NEDA included absence of Scripps-assessed moderate and/or severe relapses (NEDA-2; OR: 3.55; *p* < 0.001) or the absence of Scripps-assessed severe relapses (NEDA-3; OR: 3.59; *p* < 0.001) rather than absence of all protocol-defined relapses (OR: 2.88; *p* = 0.006) (Fig. 6).

## Discussion

The findings from these post hoc analyses over Year 1 from the PRISMS study demonstrated that both doses of IFN β-1a SC tiw therapy had significant, early benefits on clinical, radiological, and NEDA endpoints compared with placebo. These results are consistent with the findings at 2 years in PRISMS [1].

In the analyses presented here, IFN β-1a SC tiw had sustained benefits on both clinical and radiological endpoints, with a statistically significant difference in time to first relapse between IFN β-1a SC tiw treatment groups and placebo, seen as early as Month 3 and continuing up to Year 1. Both doses of IFN β-1a SC tiw significantly reduced the risk of 3-month confirmed disability progression up to 1 year compared with placebo. In addition, improvements were seen as early as Month 2 for radiological endpoints and were maintained at Months 6, 9, and 12. Moreover, the results of prespecified subgroup analyses indicate that the effect of IFN β-1a SC tiw is consistent across a broad range of patient populations, regardless of baseline disease characteristics.

The finding of a treatment benefit for interferon therapy as early as Month 2 for radiological endpoints is noteworthy, as the modified Rio score in current use predicts response or nonresponse to interferon therapy based on MRI results after a full year [8]. It should be considered that the current analysis did not attempt to predict subsequent clinical responses based on MRI at 2 months, and that it is relatively uncommon to conduct MRI as often as every month and thus datasets used to predict clinical response based on later MRI results can be larger than our sample [9]. It has been recommended to “re-baseline” patients taking interferon therapy at 3–6 months for purposes of estimating future NEDA status [10]; although benefit was shown in this study as early as Month 2 we are not able to say that this would be a more efficient way of estimating future disease status in clinical practice.

Disease activity in the placebo arms of modern MS clinical trial populations has fallen due to changing diagnostic criteria, trial enrollment criteria, and endpoint definitions [11, 12]. For example, compared with PRISMS, more stringent definitions of relapse (e.g. a requirement for EDSS score changes in order for relapses to be confirmed) have been increasingly used in subsequent clinical trials [11, 13]. Therefore, we also assessed the impact of relapse severity on the proportion of patients achieving the composite efficacy endpoints. More patients taking IFN β-1a SC tiw were able to achieve no evidence of clinical disease activity and NEDA status compared with placebo, regardless of relapse severity.

Finally, the early effect of IFN β-1a SC tiw treatment is consistent and maintained in the long-term follow-up. The early benefit of IFN β-1a SC tiw treatment was maintained through 4 years and in the long-term follow- up in this patient population with highly active disease at baseline [2, 14]. Through 15 years in PRISMS, patients with higher cumulative dose exposure and longer duration on treatment experienced better clinical outcomes [14].

## Conclusions

IFN β-1a SC tiw treatment had significant early benefits on clinical and radiological endpoints; efficacy was also confirmed using varying definitions of NEDA. Finally, the efficacy of IFN β-1a SC tiw across patient subgroups was consistent with effects seen in the overall treatment population.

## Additional files

Additional file 1: **Table S1.** Baseline characteristics of patients in the PRISMS-2 study (adapted from PRISMS Study Group. Lancet. 1998;352:1498–504). (DOCX 15 kb)

Additional file 2: **Figure S1.** Incremental quarterly relapse count over 1 year. (PDF 145 kb)

Additional file 3: **Figure S2.** Post hoc analysis of MRI endpoints. (PDF 170 kb)

## Abbreviations

ARR: Annualized relapse rate
BOD: Burden of disease
CI: Confidence interval
EDSS: Expanded Disability Status Scale
Gd+: Gadolinium-enhancing
IFN: Interferon
MRI: Magnetic resonance imaging
MS: Multiple sclerosis
NEDA: No evidence of disease activity
NRS: Neurological Rating Scale
OR: Odds ratio
PD: Proton density
RRMS: Relapsing–remitting multiple sclerosis
SC: Subcutaneously
tiw: Three times weekly
