# Supplementary material for: Effect of interferon beta-1a subcutaneously three times weekly on clinical and radiological measures and no evidence of disease activity status in patients with relapsing–remitting multiple sclerosis at year 1
Source: BMC Neurol. 2018 Sep 14;18:143. doi: 10.1186/s12883-018-1145-x (PMC6137887; doi:10.1186/s12883-018-1145-x)
Supplement: Supplementary file 1 — Table S1. Baseline characteristics of patients in the PRISMS-2 study (adapted from PRISMS Study Group. Lancet. 1998;352:1498–504). (DOCX 15 kb) [file 12883_2018_1145_MOESM1_ESM.docx]

**Additional file 1: Table S1** Baseline characteristics of patients in the PRISMS-2 study (adapted from PRISMS Study Group. Lancet. 1998;352:1498-504)

| Characteristic | Placebo  (*n* = 187) | IFN β-1a 22 μg SC tiw (*n* = 189) | IFN β-1a 44 μg SC tiw (*n* = 189) | Total  (*N* = 560) |
| --- | --- | --- | --- | --- |
| Age, median (IQR) | 34.6  (28.8–40.4) | 34.8  (29.3–39.8) | 35.6  (28.4–41.0) | 34.9  (29.1–40.4) |
| Sex, male/female, % | 25/75 | 33/67 | 34/66 | 31/69 |
| MS duration, years (median [IQR]) | 4.3  (2.4–8.4) | 5.4  (3.0–11.2) | 6.4  (2.9–10.3) | 5.3  (2.8–10.0) |
| Relapses in previous 2 years | | | | |
| Number of relapses, (mean [SD]) | 3.0 (1.3) | 3.0 (1.1) | 3.0 (1.1) | 3.0 (1.2) |
| Patients with score, % | | | | |
| 2 relapses | 41 | 43 | 40 | 41 |
| 3 relapses | 36 | 29 | 34 | 33 |
| ≥4 relapses | 23 | 28 | 26 | 26 |
| EDSS score at baseline, mean (SD) | 2.4 (1.2) | 2.5 (1.2) | 2.5 (1.3) | 2.5 (1.2) |
| Patients with EDSS score, % |  |  |  |  |
| ≤1.5 | 33 | 30 | 31 | 32 |
| 2.0–2.5 | 28 | 27 | 26 | 27 |
| 3.0–3.5 | 24 | 24 | 26 | 24 |
| ≥4.0 | 15 | 19 | 17 | 17 |

EDSS: Expanded Disability Status Scale; IFN β-1a: interferon beta-1a; IQR: interquartile range; MS: multiple sclerosis; SC: subcutaneously; tiw: three times weekly.
