# Supplementary material for: Effect of interferon beta-1a subcutaneously three times weekly on clinical and radiological measures and no evidence of disease activity status in patients with relapsing–remitting multiple sclerosis at year 1
Source: BMC Neurol. 2018 Sep 14;18:143. doi: 10.1186/s12883-018-1145-x (PMC6137887; doi:10.1186/s12883-018-1145-x)
Supplement: Supplementary file 2 — Figure S1. Incremental quarterly relapse count over 1 year. (PDF 145 kb) [file 12883_2018_1145_MOESM2_ESM.pdf]

Additional file 2

Incremental quarterly relapse count over 1 year

Patients treated with placebo had numerically higher numbers of relapses than patients treated with IFN β-1a SC tiw over each 3-month incremental period during Year 1 of the PRISMS trial.

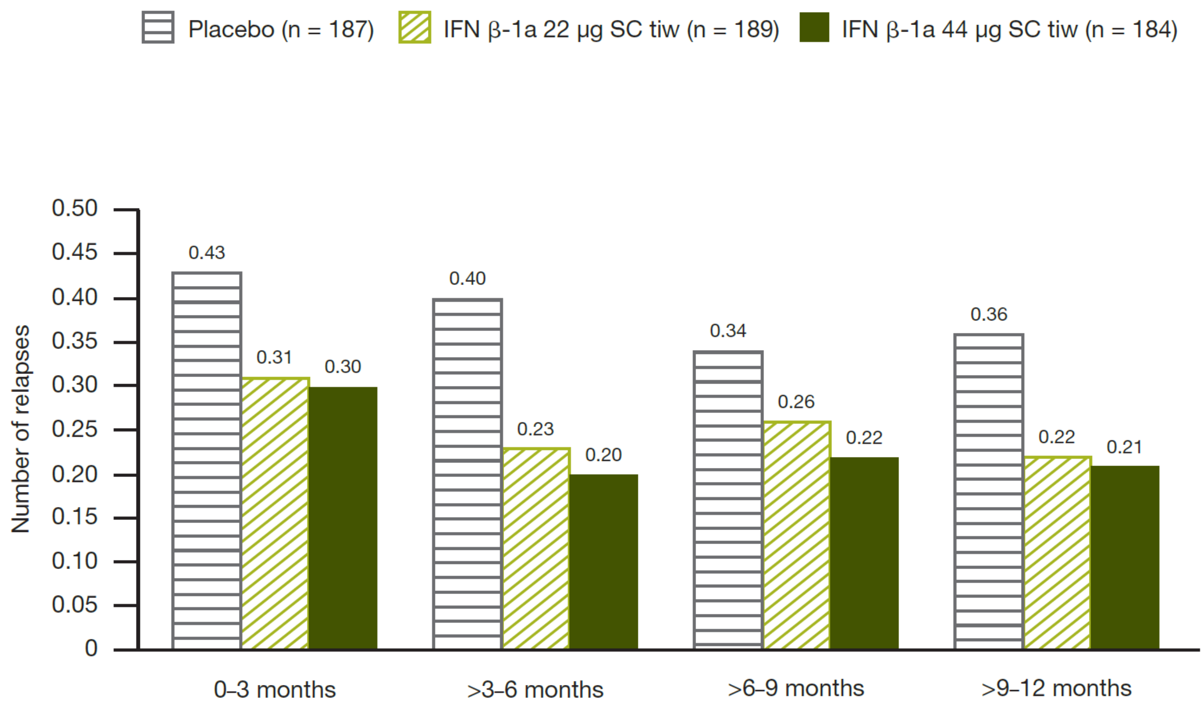

Fig. S1 Mean number of clinical relapses over each 3-month interval.

IFN β-1a: interferon beta-1a; SC: subcutaneous; tiw: three times weekly.
