# Supplementary material for: Effect of interferon beta-1a subcutaneously three times weekly on clinical and radiological measures and no evidence of disease activity status in patients with relapsing–remitting multiple sclerosis at year 1
Source: BMC Neurol. 2018 Sep 14;18:143. doi: 10.1186/s12883-018-1145-x (PMC6137887; doi:10.1186/s12883-018-1145-x)
Supplement: Supplementary file 3 — Figure S2. Post hoc analysis of MRI endpoints. (PDF 170 kb) [file 12883_2018_1145_MOESM3_ESM.pdf]

Additional file 3

Post hoc analysis of MRI endpoints

Significantly greater proportions of patients receiving interferon beta-1a (IFN β-1a) 44 µg subcutaneously (SC) three times weekly (tiw) were free of gadolinium-enhancing (Gd+) lesions from Months 2–9 compared with placebo (Month 2: 81.3% vs. 48.4%, $p = 0.0002$; Month 9: 88.2% vs. 52.3%, $p < 0.0001$; Supplementary Fig. 2A). Cumulative mean numbers of Gd+ lesions per patient per scan were significantly lower in the IFN β-1a 44 and 22 µg SC tiw groups compared with placebo from Months 2–9 (Supplementary Fig. 2B). IFN β-1a 44 and 22 µg SC tiw were also associated with significantly fewer mean active T2 lesions per patient per scan compared with placebo beginning Month 3 through to Month 9 ($p < 0.05$) and with fewer mean combined unique active lesions from Months 2–9 ($p < 0.01$).

a)

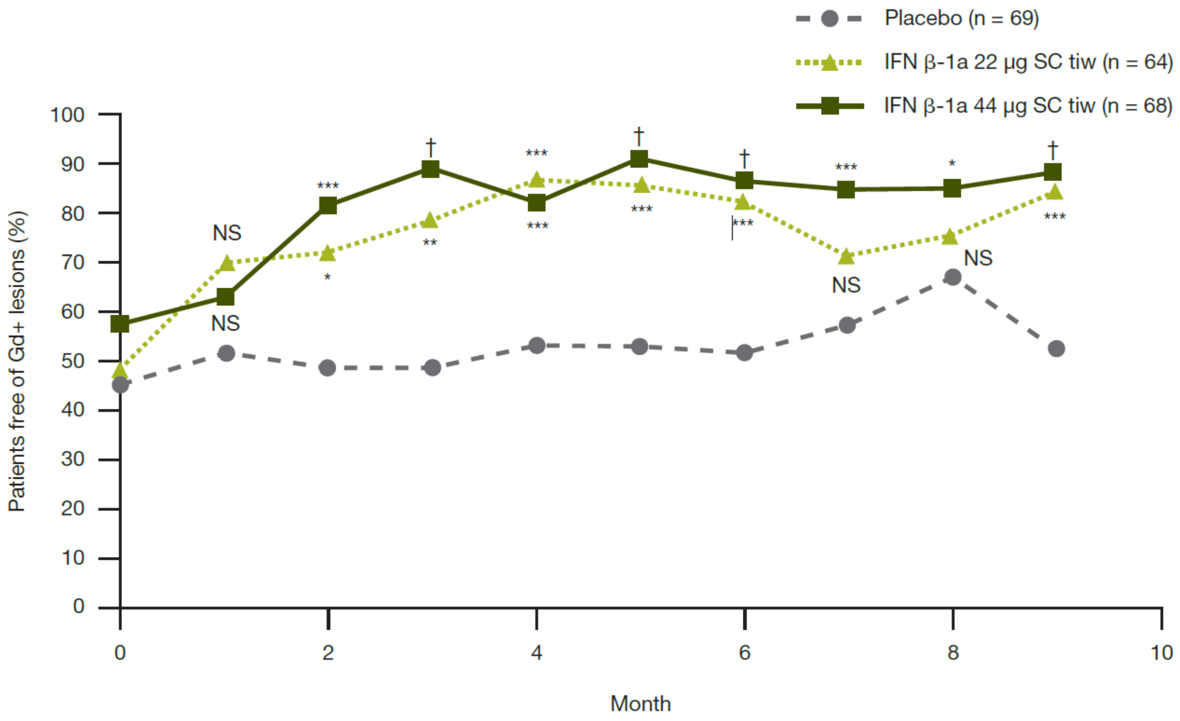

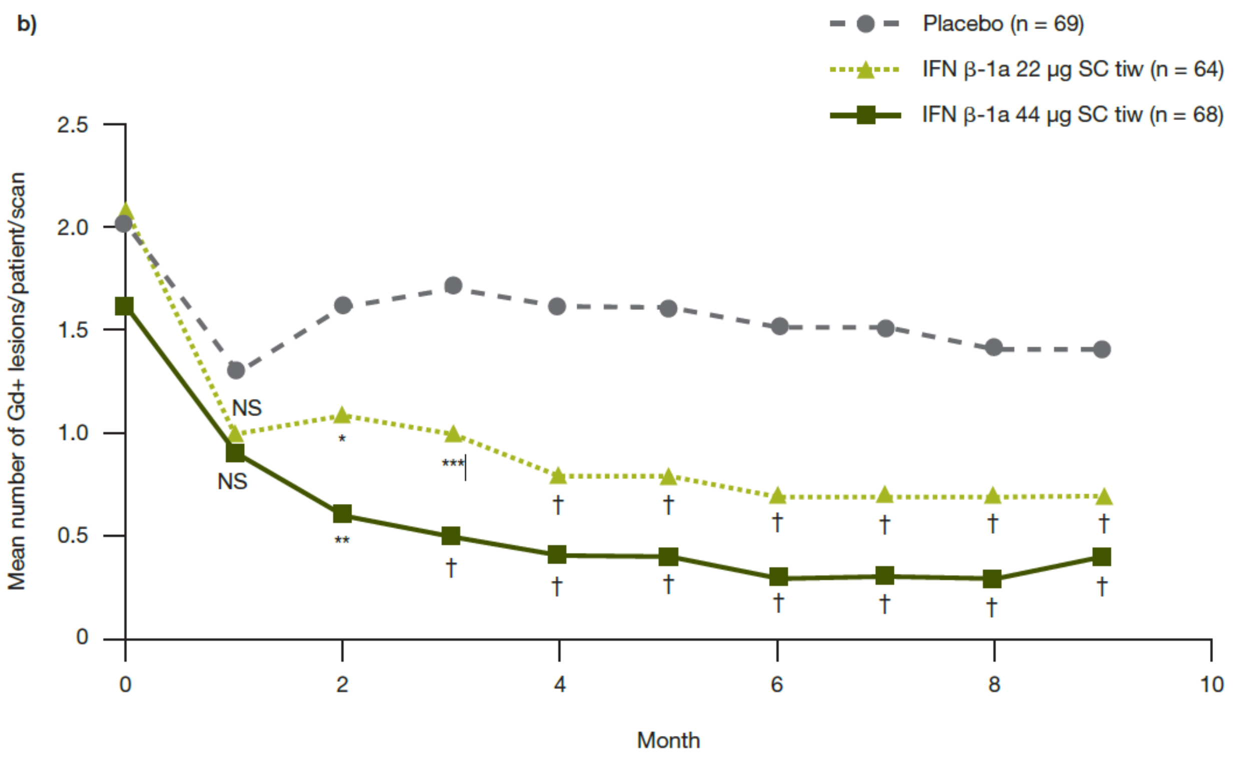

Fig. S2 Effect of IFN β-1a SC tiw on MRI activity on Gd+ lesions over the first 9 months of treatment (frequent-MRI cohort from PRISMS; *n* = 205). **(a)** Proportion of patients free of Gd+ lesions. Based on logistic regression adjusting for number of corresponding lesions at baseline. **(b)** Cumulative mean number of Gd+ lesions per patient per scan.

[a]Comparisons of cumulative mean number of lesions from baseline to Month 9 per patient per scan were based on a negative binomial model adjusting for the corresponding number of lesions at baseline with the log of the number of scans as the offset variable. Comparisons of the proportion of patients free of Gd+ lesions at each monthly scan from baseline to Month 9 were based on a logistic regression adjusting for the corresponding number of lesions at baseline.

*$p < 0.05$; **$p < 0.01$; ***$p < 0.001$; †$p < 0.0001$ compared with placebo.

[a]Cumulative mean was calculated across all scans (i.e. cumulative mean number of lesions were divided by total number of scans received up to each time point).

Gd+: gadolinium-enhancing; IFN β-1a: interferon beta-1a; MRI: magnetic resonance imaging; NS: not significant vs. placebo; SC: subcutaneously; tiw: three times weekly.
